# Mechanisms underlying the dynamic changes in tannins associated with food processing and plant growth

**DOI:** 10.1007/s11418-025-01925-3

**Published:** 2025-06-25

**Authors:** Takashi Tanaka

**Affiliations:** Graduate School of Biomedical Sciences, Nagasaki University, 1-14 Bunkyo-Machi, Nagasaki, 852-8521 Japan

**Keywords:** Tannin, Polyphenol, Catechin, Proanthocyanidin, Ellagitannin, Black tea

## Abstract

This review describes the chemical mechanisms behind the structural changes in selected tannins associated with food processing and plant growth. Both the artificial removal of astringency from persimmon fruits and production of hydrophobic procyanidins in cinnamon bark occur via the condensation of proanthocyanidin A-rings with aldehydes. The production of black tea thearubigins from monomeric catechins and the oligomerization of epigallocatechin-3-*O*-gallate (EGCg) by autoxidation have been explained via the addition of catechin A-rings to B-ring* o*-quinones. These reactions can be ascribed to the nucleophilic properties of the A-ring methine carbons. Meanwhile, the oxidative B-B coupling of EGCg first produces a quinone dimer, dehydrotheasinensin A (DTSA), and subsequent reduction yields theasinensin A with a bis-pyrogallol structure. The structural similarity of DTSA to ellagitannin dehydrohexahydroxydiphenoyl (DHHDP) groups led us to propose a new hypothesis concerning ellagitannin biosynthesis, in which the oxidative coupling of two galloyl groups first produces a DHHDP group, and subsequent reduction yields a hexahydroxydiphenoyl (HHDP) group. In fact, the DHHDP-bearing ellagitannin in the young leaves of *Triadica sebifera* is reduced to the corresponding HHDP ester as the leaves grow. Additionally, CuCl_2_ oxidation of gallic acid esters and 1,2,3,4,6-pentagalloyl-*β*-d-glucose yields DHHDP esters rather than HHDP esters. In contrast, in the young leaves of a Japanese oak tree, ellagitannin vescalagin is oxidized regioselectively as the leaves grow; this oxidation reaction is related to the autoxidation of vescalagin in oak barrels during whisky aging. Furthermore, this review discusses the immobilization of vescalagin in heartwood.

**Graphical abstract:**

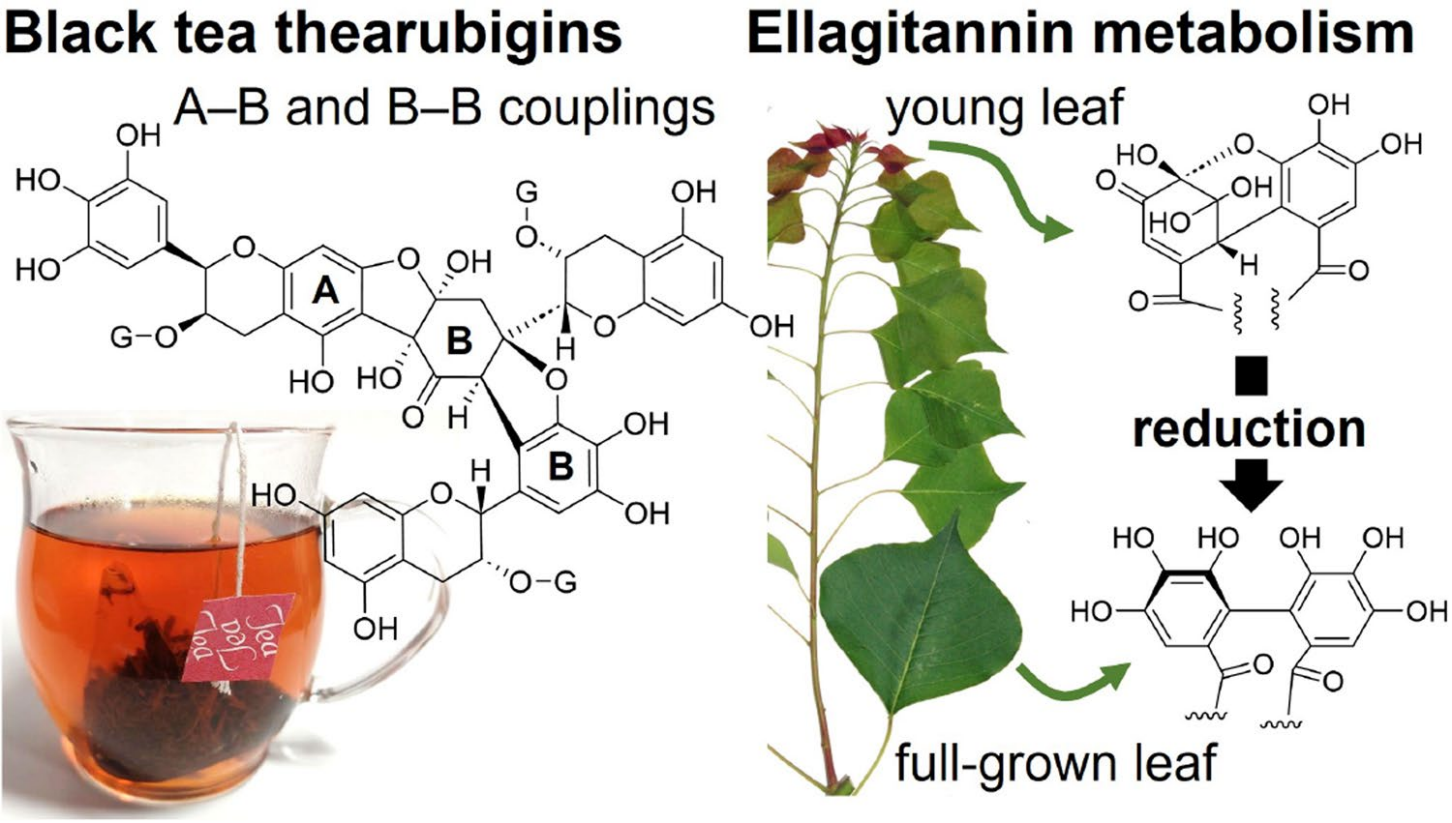

## Introduction

Since the 1980s, epidemiological and biological studies have revealed the protective effects of polyphenol-rich foods such as wine and tea against various diseases, including cancer and cardiovascular diseases [1–5]. Plant polyphenols have attracted significant attention from researchers in the life sciences. Tannins are a group of polyphenols that can aggregate proteins, causing astringency and bitterness in the oral cavity and rendering proteins nutritionally unavailable. Therefore, the presence of tannins in food is undesirable for humans [6]. Various methods have been developed to decrease tannin contents in foods or to remove their unpleasant taste. For example, black tea is produced by the mechanical crushing of withered tea leaves. This process mixes astringent galloyl catechins with oxidation enzymes, and the subsequent aeration of leaves decreases the content of galloyl catechins and produces a complex mixture of oxidation products characteristic of black tea. As observed in black tea production, tannins and related polyphenols are chemically altered during food processing, such as cutting, drying, heating, and fermentation, because they are susceptible to oxidation and/or hydrolysis. However, the mechanism behind these reactions has not yet been clarified because, in many cases, such reactions produce inseparable mixtures of oligomeric products. In some cases, the products are insoluble and non-extractable. Therefore, methodologies commonly used in pure natural product chemistry, namely chromatographic purification and structural determination using spectroscopic techniques, are sometimes ineffective in elucidating the complex chemical phenomena involved in these reactions. In catechin oxidation during black tea manufacturing, the production mechanisms for dimeric products have been studied well; however, these mechanisms do not necessarily represent oligomerization mechanisms [7]. Some structural changes in tannins are associated with plant growth. In leaves of a Japanese oak (*Quercus glauca*), ellagitannins are detected only in young leaves and not in fully grown ones. Our research group has long studied the chemistry of tannins, and this review focuses on the reaction mechanisms of the structural changes in selected tannins associated with food processing and plant growth.

### Chemical properties of catechin A- and B-rings

**Fig. 1:**
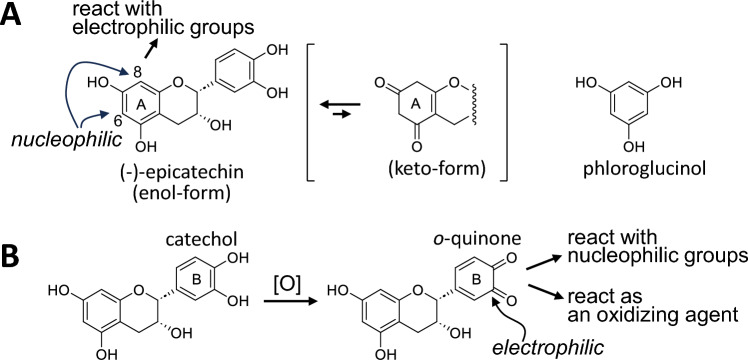
Reactivities of epicatechin. Keto–enol tautomerization of the A-ring and structure of phloroglucinol (**A**). Oxidation of the B-ring and reactivities of the *o*-quinone (**B**)

We first focus on the basic reactivities of catechins and procyanidins (condensed tannins), because they are related to the major topics discussed in this review. The two benzene rings in the catechin skeleton are referred to as the A- and B-rings, respectively (Fig. 1). The A-ring has a 1,3,5-trioxygenated benzene structure similar to that of phloroglucinol. The two methine carbons of the A-ring, C-6 and C-8, are electron-rich compared to other aromatic carbons because the A-ring can be regarded as an enol form of keto–enol tautomerization. Therefore, C-8 and C-6 react with electrophilic groups such as aldehydes and *o*-quinones [8]. This property of the catechin A-ring is similar to that of the A-ring in proanthocyanidins.

The B-rings of most natural catechins and proanthocyanidins have two or three vicinal phenolic hydroxy groups and are referred to as catechol- and pyrogallol-type B-rings, respectively. These catechin B-rings are often oxidized to *o*-quinones, which are electrophilic and tend to react with electron-rich carbons such as C-6 and C-8 of the catechin A-rings [9]. In addition, the *o*-quinone of the catechol-type B-ring behaves as an oxidizing agent and oxidizes pyrogallol-type B-rings with a relatively low redox potential [10].

## Removal of astringency from persimmon fruits

**Fig. 2:**
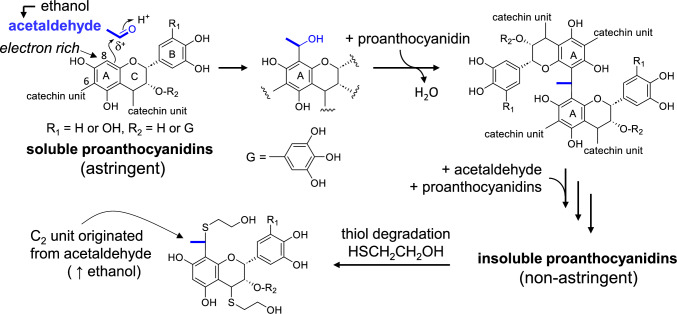
Insolubilization of persimmon proanthocyanidins by condensation with acetaldehyde and thiol degradation of proanthocyanidins insolubilized in fruits by anaerobic treatment with 30% EtOH. Blue bold lines represent the units originating from acetaldehyde

Among the many persimmon cultivars, astringent-type cultivars have a strong astringent taste due to the presence of galloyl proanthocyanidins. This astringency can be artificially removed by treating the persimmon fruits with carbon dioxide or aqueous ethanol in sealed polyethylene bags. These anaerobic treatments induce the production of acetaldehyde in fruits [11], which reacts nonenzymatically with the C-8 or C-6 carbons of proanthocyanidin A-rings (Fig. 2). The C_2_ unit connected to the A-ring reacts with another proanthocyanidin molecule to yield a proanthocyanidin dimer. Further reactions of the dimer with acetaldehyde crosslink the proanthocyanidins to make the molecules insoluble in tannin cells, resulting in the removal of astringency from the fruit flesh. This mechanism was chemically confirmed by the direct application of thiol degradation to fruits treated with aqueous ethanol or deuterated ethanol in sealed polyethylene bags [12]. In this experiment, the thiol group of 2-mercaptoethanol attacked the benzyl methine carbons to cleave interflavan C–C bonds to produce monomeric catechin fragments (Fig. 2). Among the products, monomers bearing a C_2_ unit originating from acetaldehyde were identified. Thus, the incorporation of acetaldehyde into the insolubilized proanthocyanidins was confirmed. Acetaldehyde production in fruits depends on the cultivar [13, 14]. In cultivars that do not produce acetaldehyde, tannin insolubilization involves complex formation with soluble pectins, which reduces astringency during tree maturation [15, 16].

### Hydrophobic proanthocyanidins of cinnamon bark

**Fig. 3:**
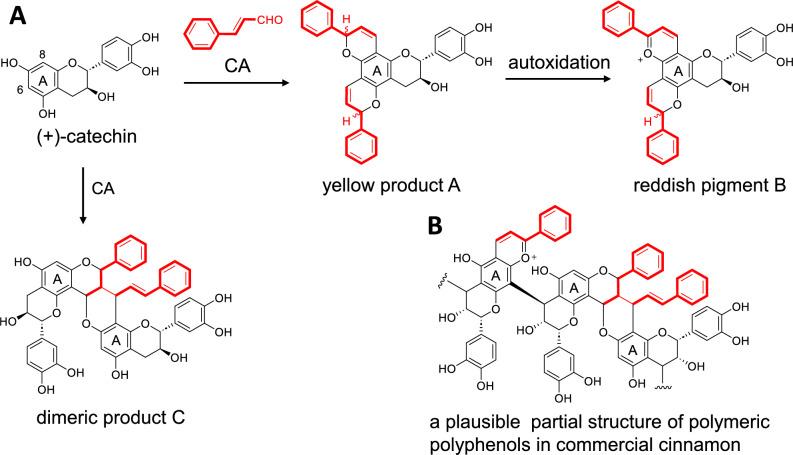
Reaction of ( +)-catechin with cinnamic aldehyde (**A**) and a plausible partial structure of polymeric polyphenols in commercial cinnamon (**B**). *CA* cinnamic aldehyde. Partial structures depicted in red bold lines are the units originated from cinnamic aldehyde

Procyanidins in Japanese cinnamon bark are composed of epicatechin and catechin units [17]. When fresh bark is peeled off from the wood, colorless proanthocyanidins turn red within a few hours because of their non-enzymatic reactions with cinnamic aldehyde. This mechanism was proposed based on model experiments using a simple catechin monomer. When (+)-catechin is mixed with cinnamic aldehyde at room temperature, a complex mixture containing reddish pigments is produced [18]. Repeated experiments under various conditions revealed that the initial product contained the unstable yellow product A (Fig. 3A). The product was oxidized by ambient oxygen to produce an unstable reddish pigment B with an anthocyanidin-like structure. Another experiment yielded several diastereomers of the dimeric product C. These results suggest that cinnamic aldehyde also reacts with procyanidin A-rings in cinnamon bark. This was supported by MALDI-TOF-MS of a mixture of a dimeric procyanidin and cinnamic aldehyde, which showed oligomerization by cross-linking between the compounds. Furthermore, ^13^C NMR spectroscopy of the polymeric polyphenol fraction obtained from commercial cinnamon bark showed signals corresponding to the monosubstituted benzene ring originating from cinnamic aldehyde. These results strongly suggest that cinnamon procyanidins are modified by reaction with cinnamic aldehyde during harvesting and drying. This explains why the polymeric procyanidins in commercial cinnamon bark are hydrophobic compared to usual ones (Fig. 3B). The cinnamon polymeric polyphenols used in this study were purified by size-exclusion chromatography using a Sephadex LH-20 column with aqueous acetone containing a high concentration of urea [19]. This method, combined with adsorption chromatography, is useful for separating oligomeric polyphenols from low-molecular-weight polyphenols.

### Black tea thearubigins: oxidative oligomerization of tea catechins

**Fig. 4:**
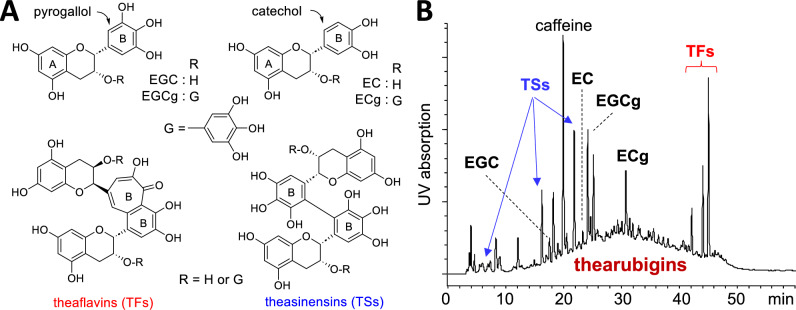
Structures of representative black tea polyphenols (**A**) and HPLC profile of the 60% EtOH extract of a commercial black tea (**B**)

Black tea is produced using the following processes. Harvested fresh tea leaves are first withered to control moisture and then mechanically twisted or crushed to break the leaf cells. This process mixes tea catechins with oxidation enzymes, and subsequent aeration supplies oxygen to oxidize catechins. The enzymes are then inactivated by heating during the final drying process. Theaflavins [20] and theasinensins [21] (Fig. 4A) are representative catechin oxidation products present in black tea [7]. In addition to these well-characterized catechin dimers, reddish-brown pigments composed of oligomeric products are produced. These pigments were designated thearubigins in the late 1950s [22–24]; however, the composition and structure of these components have not yet been clarified. Thearubigins are the most important dietary polyphenols for humans because black tea accounts for 70–80% of world tea production, and thearubigins constitute up to 60% of the solids in black tea infusions [25]. In addition, recent studies have reported biological activities of thearubigins [26, 27], such as the inhibition of lipase and amylase [28] and stimulation of exercise training-induced improvement in endurance capacity in mice [29–31].

In HPLC analysis, thearubigins are detected as a Gaussian-shape broad hump on the baseline (Fig. 4B). In previous studies, pure thearubigins that are free of catechin monomers and dimers were separated by the combined use of adsorption and size-exclusion chromatography with Sephadex LH-20 and Diaion HP20 columns [19, 28, 32]. However, the structural determination of individual thearubigin components is virtually impossible. Therefore, we investigated the production mechanisms of thearubigin components [32]. Green tea polyphenols are mainly composed of four monomeric catechins: ( −)-epigallocatechin (EGC), ( −)-epigallocatechin-3-*O*-gallate (EGCg), ( −)-epicatechin (EC), and ( −)-epicatechin-3-*O*-gallate (ECg) (Fig. 4A). Since the former two catechins with a pyrogallol-type B-ring account for approximately 70% of the total tea catechins, oxidative coupling of EGCg and EGC must be important in thearubigin formation. The ^13^C NMR spectrum of pure thearubigins showed signals corresponding to the catechin skeleton and galloyl groups except for the signals assignable to the methine carbons of pyrogallol-type B-rings (δ_C_ 106–108). The absence of the B-ring methine signals indicated that the pyrogallol-type B-ring is responsible for oligomerization of the four tea catechins [7, 28, 32].

**Fig. 5:**
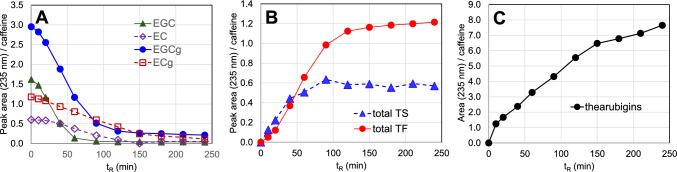
Time-course of HPLC peak area of black tea polyphenols during a model experiment using lyophilized fresh tea leaves. Enzymatic oxidation was initiated by adding H_2_O at 0 min. The peak areas are relative value to endogenous caffeine. Monomeric catechins (**A**), total theaflavins (TF) and total theasinensins (TS) (**B**), and thearubigins (**C**)

To clarify the dynamics of thearubigin production in tea leaves, the time course of catechin oxidation was examined using powder of lyophilized fresh tea leaves [33]. The powder retained enzyme activity, and the addition of H_2_O initiated the same catechin oxidation as that in crushed fresh leaves. HPLC analysis of the wet leaf powder showed a rapid decrease in EGCg and EGC contents (Fig. 5A), accompanied by an increase in theasinensins and theaflavins (Fig. 5B), which are oxidation products of EGC(g) [32]. The concentration of thearubigins increased from the beginning of the reaction and continued to increase after theaflavin and theasinensin contents reached a plateau (Fig. 5C).

In addition to theaflavins and theasinensins, various minor catechin dimers and trimers have been identified in black tea or reaction mixtures of model experiments [7, 34–37]. However, our investigation including various model experiments suggested that production of oligomers by elongation of the dimers by further intermolecular couplings, such as B-galloyl coupling, were estimated to be very limited [7]. Therefore, the increase in thearubigin contents from the beginning of catechin oxidation in the above time-course experiment (Fig. 5C) suggested the presence of another reaction mechanism for thearubigin formation [32].

**Fig. 6:**
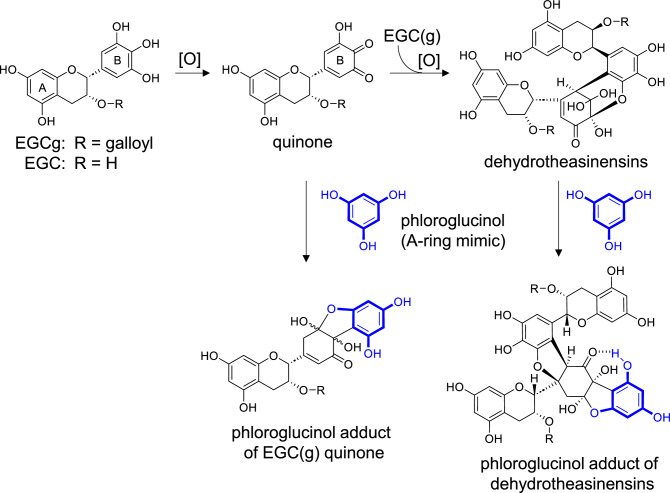
Reactions of pyrogallol-type catechins in fresh green tea leaves crushed with phloroglucinol

The enzymatic oxidation of EC alone produces dimers and trimers generated by oxidative A-B couplings [9, 10, 38]. The products retain the intact A- and B-rings, and further A-B couplings yield a complex mixture of oligomers. We assumed that similar A-B couplings also occurred during the oxidation of pyrogallol-type catechins. Based on this assumption, fresh tea leaves were crushed with phloroglucinol, an A-ring mimic possessing the same reactivity as catechin A-rings (Fig. 1A) [32]. This experiment yielded the phloroglucinol adducts of EGC(g) quinone and dehydrotheasinensins (Fig. 6). Dehydrotheasinensins are EGC(g) quinone dimers that accumulate in crushed fresh tea leaves and are converted into theasinensins when the leaves are heated and dried during the final stage of black tea production (vide infra) [39, 40].

**Fig. 7:**
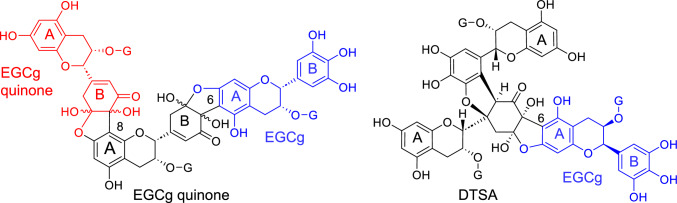
Possible partial structures of thearubigins formed by the A-B coupling mechanism

Production of the phloroglucinol adducts strongly suggested that the *o*-quinones of pyrogallol-type catechins and dehydrotheasinensins bind to the A-rings of catechins or catechin oxidation products, such as theasinensins and theaflavins. Based on these results, two possible partial structures of thearubigins are shown in Fig. 7. The structures shown in Fig. 7 retain the reactive A-rings, which enable further elongation of the molecules by couplings with B-ring *o*-quinones. This observation was supported by the fact that longer aeration of the tea leaves increased the concentrations of thearubigins with larger molecular weights [32]. Our previous studies suggest that the enzymes catalyze only the production of *o*-quinones, and the subsequent reactions are non-enzymatic. Moreover, the A-B couplings are presumed to be non-regio- and stereoselective [7, 10]. These features explain the compositional complexity of the thearubigins. Therefore, the A-B coupling mechanism is the most likely to be the major mechanism for thearubigin production. Furthermore, a study on the autoxidation of EGCg suggested that further structural changes in the A-B linkages shown in Fig. 7 possibly occur (vide infra) [41]. Size-exclusion HPLC using a calibration curve obtained by commonly used polystyrene standards estimated the molecular weights of thearubigins to be 20000–30000 (about 60-mers of tea catechins); however, in the same HPLC experiment, comparison of the retention times of dimeric and trimeric ellagitannin standards (MW 1870 and 2804, respectively) [42] suggested that the major components of thearubigins correspond with tetramers–hexamers of tea catechins [32]. This is more likely considering the physicochemical properties of the standards and thearubigins.

### Autoxidation of epigallocatechin gallate in neutral aqueous solution

**Fig. 8:**
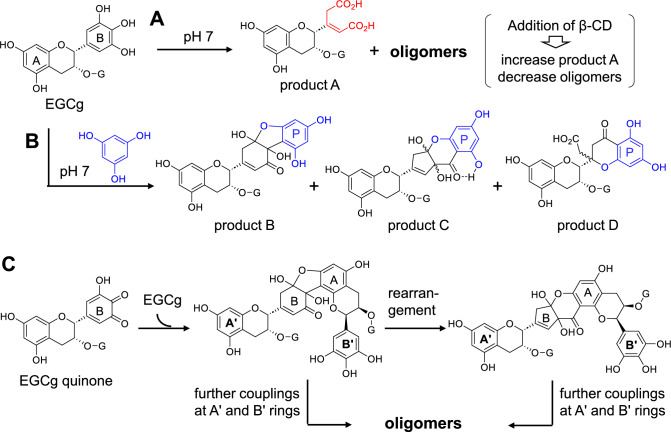
Autoxidation of epigallocatechin-3-*O*-gallate (EGCg) in neutral buffer solution (pH 7). Production of a monomeric product A and oligomers (**A**). Production of products **B**–**D** by treatment with phloroglucinol (**B**), and a plausible mechanism for oligomerization during the autoxidation of EGCg (**C**). *P* aromatic rings originating from phloroglucinol

Many health benefits of tea catechins are attributable to their antioxidant activity, which implies that they are highly susceptible to oxidation. Tea catechins are oxidized by dissolved oxygen in neutral or alkaline solutions (pH > 6), a process referred to as autoxidation [43, 44]. Autoxidation occurs during food preservation and the evaluation of biological activities by in vitro cell culture [45, 46]. Therefore, determining the chemical information about the autoxidation products is important. Previous studies on the autoxidation of EGCg showed the production of monomeric products generated by the oxidative cleavage of the B-ring, such as product A in Fig. 8A [47, 48], as well as dimeric products generated by B-B coupling, including theasinensin A (Fig. 4) [47–49]. However, the major products were complex mixture of oligomers. To elucidate the oligomerization mechanism, the changes in EGCg in a pH 7 buffer solution were examined under various conditions. *β*-Cyclodextrin (β-CD) forms a 1:1 complex with EGCg, in which the A-ring and a portion of the C-ring are included from the wide secondary hydroxyl group side of the β-CD cavity, and the B-ring and galloyl groups are left outside the cavity [50]. Interestingly, the addition of β-CD to the reaction mixture of the EGCg autoxidation decreased the contents of the oligomers and increased the amount of monomeric products [41]. These results indicate that the A-ring of EGCg participates in oligomerization. Assuming that the oligomers were produced by A-B couplings, the autoxidation of EGCg was examined in the presence of phloroglucinol as an A-ring mimic. The experiment yielded products B, C and D (Fig. 8B), and their structures strongly suggested that oligomerization proceeded via A-B coupling [41]. Although the structures B, C, and D implied that the oligomerization of EGCg proceeds via complicated mechanisms, one possible mechanism is shown in Fig. 8C. It should be emphasized that product B was the same as that obtained in the aforementioned model experiment using fresh tea leaves and phloroglucinol (Fig. 6) [32]. This suggests that the partial structures of the thearubigins illustrated in Fig. 7 can be converted to the structures related to those shown in Fig. 8C during the final heating and drying processes in black tea production. Notably, during autoxidation, oxygen molecules are reduced to hydrogen peroxide, which accounts for the bactericidal activity of catechins [43].

### Conjugation of catechin with the Strecker aldehyde of theanine

**Fig. 9:**
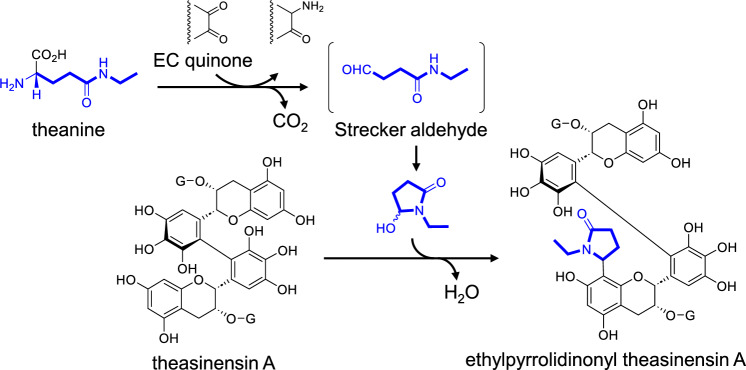
Production of theanine Strecker aldehyde and conjugation with theasinensin A

Theanine (l-γ-glutamyl ethyl amide) (Fig. 9) is an amino acid characteristic to tea plant and accounts for about 50% of the total amino acids in high-grade green tea [51, 52]. Additionally, it has been reported that Strecker aldehydes are produced from corresponding amino acids in the presence of EC *o*-quinone [53]. Since EC quinone is generated during black tea production [10], the Strecker aldehyde of theanine may be produced in black tea. A derivative of theasinensin A, with a cyclic form of theanine Strecker aldehyde, was isolated from commercial black tea (Fig. 9) [54]. Its structure was confirmed via chemical synthesis and spectroscopic investigations. Subsequently, monomeric catechins with the same *N*-ethyl-2-pyrrolidinone units were identified in various tea products [55, 56].

### Application of the reactions: development of functional catechin derivatives

**Fig. 10:**
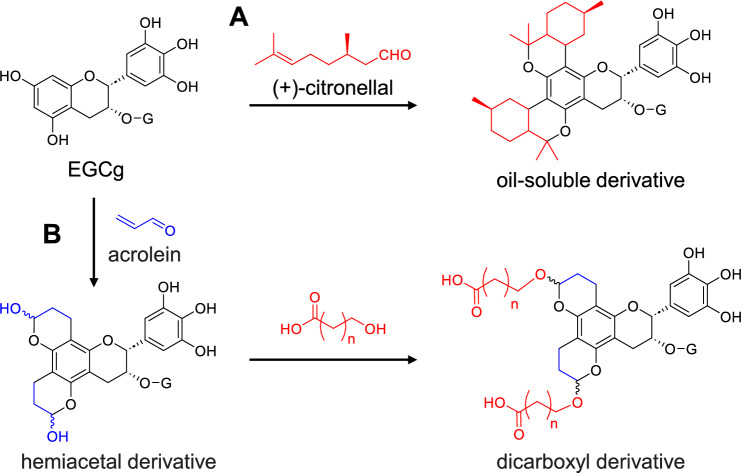
Preparation of an oil-soluble derivative (**A**) and a derivative with two carboxy groups (**B**) from EGCg

Reactions of the catechin A-rings with various aldehydes were used to prepare lipid-soluble derivatives of EGCg. For example, heating EGCg with ( +)-citronellal afforded a derivative having two monoterpene units (Fig. 10A) [57]. EGCg is practically insoluble in cooking oil; however, the derivative is highly hydrophobic (log P_*n*-octanol/water_ > 10) and exhibits radical scavenging activity in cooking oil. In addition, a bis-hemiacetal derivative prepared by the reaction with acrolein enabled the introduction of various functional groups into the EGCg A-ring (Fig. 10B). For example, treating the derivative with a ω-hydroxy carboxylic acid afforded a EGCg derivative with carboxyl groups. The derivative was further connected to magnetic beads to probe EGCg-binding proteins [58].

## Dihydrochalcone oligomers in Chinese sweet tea

**Fig. 11:**
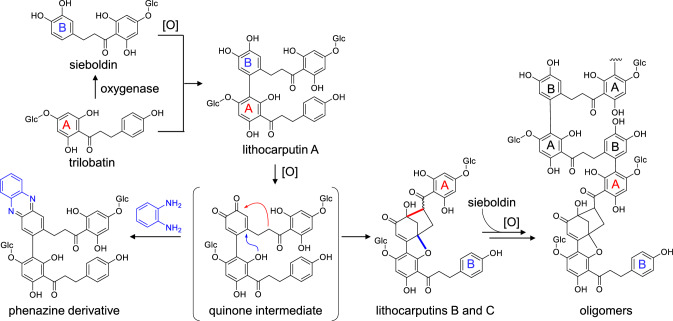
Reaction mechanism for the oxidative oligomerization of dihydrochalcone glucoside

Flavonoids with a C-4 ketone group, such as taxifolin, are more stable than catechins, because the electron-withdrawing effect of the ketone group decreases the reactivity of the A-rings. However, commercial Chinese sweet tea produced from *Lithocarpus polystachyus* leaves contains dimers and oligomers of dihydrochalcone glucosides produced by oxidative A-B coupling (Fig. 11) [59]. Trilobatin is the major dihydrochalcone glucoside in *Lithocarpus polystachyus* leaves and has a moderately sweet taste [60]. Treatment of trilobatin with polyphenol oxidase affords dimers and oligomers identical to those obtained from sweet tea [59]; the mechanism for this reaction is illustrated in Fig. 11, and the production of dimeric quinone intermediates was confirmed by isolating phenazine derivatives. The oxidation products were likely generated during harvesting or drying of the leaves.

## Microbial fermentation of green tea

**Fig. 12:**
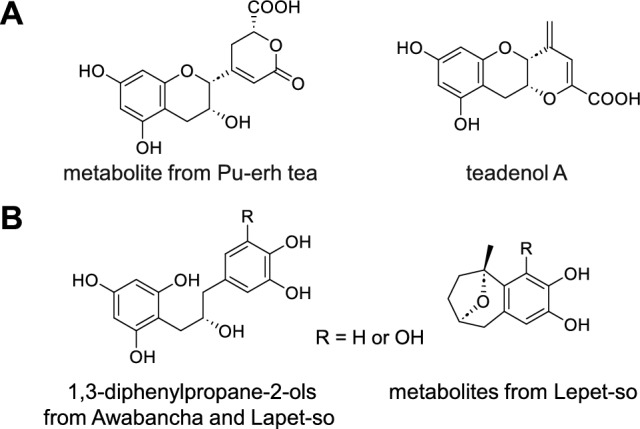
Structures of microbial metabolites of tea catechins. Aerobic fermentation (**A**) and anaerobic fermentation (**B**) (R = H or OH)

Aerobically and anaerobically fermented tea products are locally produced in East Asia. The aerobic fermentation of green tea by Aspergillus sp., such as Pu-erh tea in China and Goishicha in Japan, hydrolyzes galloyl esters, oxidizes B-rings, and oligomerizes catechins (Fig. 12A) [61–63]. In contrast, the anaerobic fermentation of green tea by Lactobacillus sp., such as Lepet-so in Myanmar and Awabancha in Japan, reductively cleaves the catechin C-ring to produce 1,3-diphenylpropane-2-ols and further degrades the A-ring (Fig. 12B) [63–65]. Notably, 1,3-diphenylpropane-2-ols are also produced as the intestinal metabolites of green tea catechins [66]. However, the mechanisms underlying the structural changes in catechins during both anaerobic and aerobic fermentation processes are not fully understood.

### Oxidative coupling of galloyl groups in ellagitannin biosynthesis

**Fig. 13:**
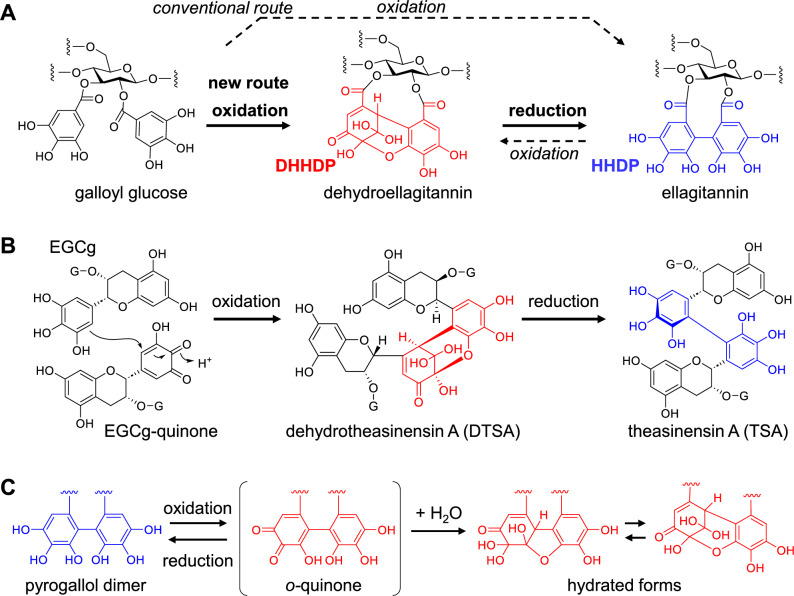
Biosynthesis of ellagitannin acyl groups (**A**). Dashed arrow: the conventional mechanism. Solid arrow: the new mechanism. Production of black tea theasinensin A (TSA) from eigallocatechin-3-*O*-gallate (EGCg) (**B**), and formation of hydrated hemiacetal structures of the DHHDP ester and dehydrotheasinensin A (DTSA) (**C**)

Ellagitannins are a group of hydrolyzable tannins biosynthesized from galloyl glucoses. The biosynthesis begins with intramolecular oxidative coupling between two galloyl groups [67, 68], which generates the most common and important ellagitannin acyl groups, namely hexahydroxydiphenoyl (HHDP) and dehydrohexahydroxydiphenoyl (DHHDP) groups (Fig. 13A). HHDP esters are the most common acyl groups in ellagitannins, and those bearing DHHDP esters are referred to as dehydroellagitannins. The DHHDP ester is an *o*-quinone of the HHDP ester and usually exists as an equilibrium mixture of hydrated hemiacetal forms [69], similar to the partial structure of dehydrotheasinensins (Fig. 13B and C) [40].

Conventionally, it has been hypothesized that HHDP esters are produced by simple oxidative coupling between two galloyl groups and that further oxidation of the HHDP group affords the DHHDP group (Fig. 13A broken allows) [70, 71]. This hypothesis was based on the compositional characteristics of hydrolyzable tannins in various plants [72] and an implicit assumption that polyphenols are susceptible to oxidation. Furthermore, the hypothesis was strongly supported by in vitro enzymatic conversion of 1,2,3,4,6-penta-*O*-galloyl-*β*-d-glucose to 1,2,3-tri-*O*-galloyl-4,6-(*S*)-HHDP glucose (tellimagrandin II), where the HHDP group was formed directly from two galloyl groups [73]. Many recent successes in total synthesis of ellagitannins have been based on the same hypothesis [74, 75]. However, we proposed a new hypothesis, in which the oxidative coupling of galloyl groups first affords the DHHDP ester, and the HHDP ester is produced by the reduction of the DHHDP ester (Fig. 13A) [76].

**Fig. 14:**
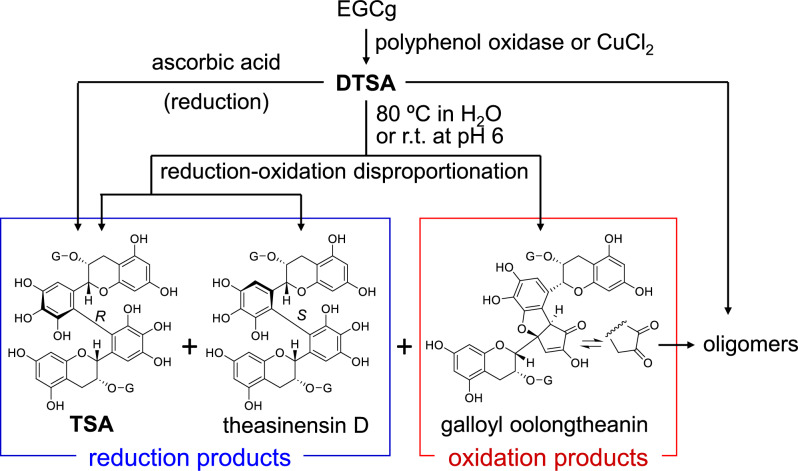
Reduction–oxidation disproportionation of dehydrotheasinensin A (DTSA) and production of theasinensin A (TSA), galloyl oolongtheanin, and oligomers

Our hypothesis is based on the structural analogy between the HHDP group and black tea theasinensins, both of which possess the same bis-pyrogallol structure (Fig. 13A, B). The production mechanism of theasinensins was established based on following evidence (Fig. 14). In black tea production, dehydrotheasinensin A (DTSA), rather than theasinensin A (TSA), is accumulated in the crushed tea leaves. DTSA is reduced to TSA when the leaves are heated and dried in the final process of black tea production [39]. In vitro treatment of EGCg with polyphenol oxidase also affords DTSA, rather than TSA [40]. Furthermore, the enzymatic oxidation is stereoselectively replicable by treating EGCg with CuCl_2_ in an aqueous solution to yield DTSA alone [77]. However, treating TSA with CuCl_2_ does not afford DTSA. Although DTSA is relatively stable under acidic conditions, it is unstable in neutral solutions (pH > 6) and undergoes non-enzymatic reduction–oxidation disproportionation even at room temperature (Fig. 14) [40]. The major products of this disproportionation reaction are reduction products TSA and its atropisomer theasinensin D (TSD) [78], accompanied by a complex mixture of oxidation products, including galloyl oolongtheanin and uncharacterized oligomers. DTSA is reduced to TSA almost quantitatively when treated with ascorbic acid [40, 77]. Such reducing agents probably participate in DTSA reduction in tea leaves during black tea production [39] because the TSD concentration in black tea is much lower than that of TSA [78].

**Fig. 15:**
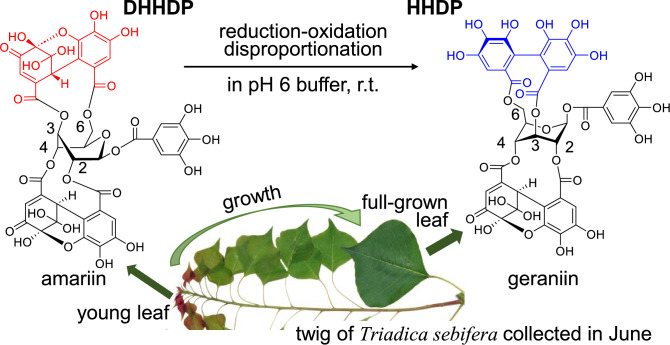
Ellagitannins present in the leaves of *Triadica sebifera* at different growth phases

A similar reduction–oxidation disproportionation was observed for ellagitannin amariin (Fig. 15). In a pH 6 buffer solution at room temperature, the concentration of amariin gradually decreased and geraniin was produced as the sole reduction product [79]. The oxidation products of the reaction have oligomeric nature and have not been characterized yet. Interestingly, in fresh leaves of *Triadica sebifera* collected in June (Fig. 15), amariin is predominant in the young leaves at the top of the twig; however, geraniin is the major tannin in the full-grown leaves of the same twig. These observations suggest that the HHDP ester is produced by DHHDP ester reduction as the leaves grow. The DHHDP ester at glucose 2,4-positions of amariin is more stable than that attached to 3,6-positions. The difference in stability can be ascribed to the differences in the flexibility of macrocyclic esters and the strain from glucose to the DHHDP esters. This is probably why majority of dehydroellagitannins bear the DHHDP ester at the 2,4-positions of ^1^C_4_-glucose [72]. However, acid hydrolysis of 1-*O*-galloyl-2,4-(*R*)-DHHDP-*β*-d-glucose (furosin) in diluted H_2_SO_4_ at 100 °C yielded ellagic acid and gallic acid, indicating that 2,4-DHHDP also undergoes reduction–oxidation disproportionation under strong conditions [79]. Similar reductive metabolism of DHHDP esters associated with plant growth was also observed in the leaves of *Carpinus laxiflora* [79], *C. japonica* [80], *Elaeocarpus sylvestris var. ellipticus* [79], and *Castanopsis sieboldii* [81].

**Fig. 16:**
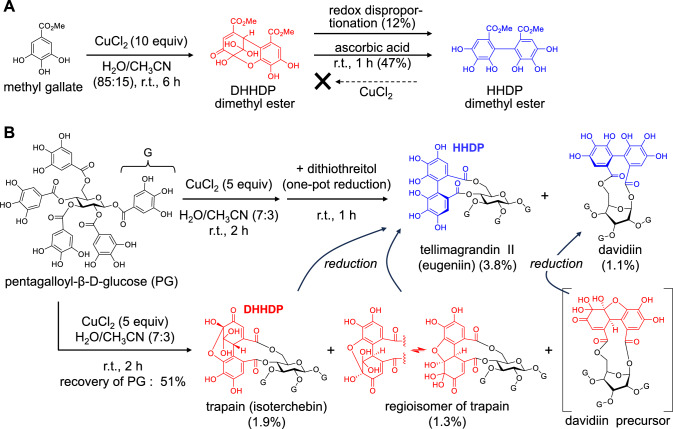
Oxidation of gallic acid methyl ester (**A**) and 1,2,3,4,6-penta-*O*-galloyl-*β*-d-glucose (**B**) with CuCl_2_ in H_2_O/CH_3_CN (7:3). Production of the davidiin precursor was confirmed by derivatization to its phenazine derivative by treating the reaction mixture with 1,2-phenylenediamine

DHHDP production from galloyl groups was further evidenced by the in vitro oxidation of methyl gallate with CuCl_2_ [82]. Although CuCl_2_ readily oxidizes the pyrogallol-type B-ring of EGCg as mentioned above [77], the pyrogallol of galloyl group resists oxidation under the same reaction conditions (Fig. 14). Therefore, a large excess of the reagent was necessary to oxidize galloyl esters, and the products were obtained in low yields. The oxidation product of methyl gallate was spectroscopically determined to be DHHDP dimethyl ester, which underwent reduction–oxidation disproportionation during chromatographic separation to yield HHDP dimethyl ester (12% from methyl gallate) (Fig. 16A). Treating the initial reaction mixture with ascorbic acid afforded the HHDP ester in higher yields (47% from methyl gallate). Meanwhile, treating the HHDP ester with CuCl_2_ under the same conditions did not afford the DHHDP ester. These results indicate that the HHDP ester is produced by the reduction of DHHDP ester. Furthermore, oxidation of 1,2,3,4,6-penta-*O*-galloyl-*β*-d-glucose with CuCl_2_, followed by one-pot reduction with dithiothreitol, afforded two natural ellagitannins, namely tellimagrandin II [syn. eugeniin, 4,6-(*S*)-HHDP-1,2,3-tri-*O*-galloyl-*β*-d-glucose] and davidiin [1,6-(*S*)-HHDP-2,3,4-tri-*O*-galloyl-*β*-d-glucose], in low yields (Fig. 16B) [83]. The precursors of tellimagrandin II were isolated from the reaction mixture of the first oxidation step and identified as trapain [syn. isoterchebin, 4,6-(*S*)-DHHDP-1,2,3-tri-*O*-galloyl-*β*-d-glucose] and its regioisomer. Although the precursor of davidiin was not isolated, its presence was confirmed by conversion to the stable phenazine derivative by treatment with 1,2-phenylenediamine. In recent ellagitannin total syntheses, HHDP biphenyl bonds have been formed by couplings between two protected galloyl groups, and the protection disables the formation of *o*-quinones [74, 75]. Conversely, the oxidation of free galloyl groups in aqueous solution first generates the *o*-quinones as observed in the oxidation of EGCg. The mechanism presented in Fig. 13A is more likely to occur in living plants rather than direct biphenyl bond formation between two galloyl groups (depicted by broken allows in Fig. 13A).

A chemotaxonomical overview of ellagitannin structures showed that taxonomically related plants tend to contain structurally related ellagitannins. This indicates that the regio- and stereoselective couplings of galloyl groups in ellagitannin biosynthesis are precisely controlled in each plant species [70, 84]. For example, DHHDP esters are widely distributed in Euphorbiaceous plants but not found in Rosaceous plants [84]. If our biogenetic hypothesis is also applicable to the HHDP esters of Rosaceous plants, enzymes involving ellagitannin biosynthesis reduce the DHHDP intermediates immediately after the coupling of galloyl groups. Notably, the change from DHHDP to HHDP esters associated with plant growth was only observed in limited plants, such as *Triadica sebifera* [79–81]. In such plants, the enzyme activity concerning DHHDP reduction may be much lower than that in Rosaceou plants.

### Seasonal change of ellagitannins in oak leaves

**Fig. 17:**
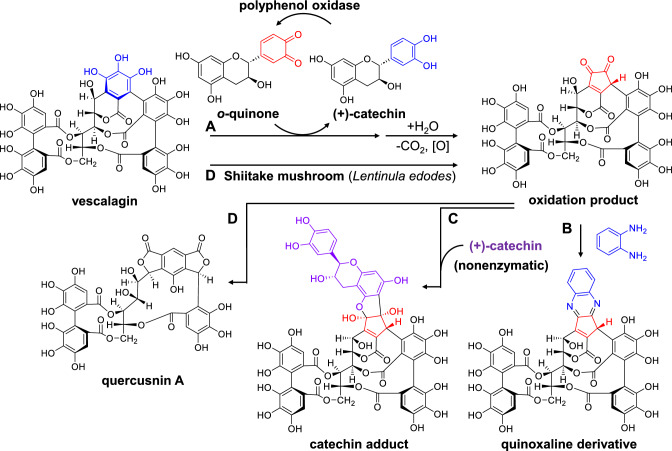
Enzymatic oxidation of vescalagin in the presence of ( +)-catechin (**A**), reactions of the oxidation product with 1,2-phenylenediamine (**B**) and ( +)-catechin (**C**), and degradation of vescalagin in Shiitake mushrooms (*Lentinula edodes*) (**D**)

In contrast to reductive metabolism, the oxidative degradation of ellagitannins occurs in the young spring leaves of *Quercus glauca*, an evergreen tree widely distributed in East Asia. Vescalagin is the major ellagitannin in young leaves (Fig. 17), and its content decreases as the leaves grow. In mature leaves, ( +)-catechins and procyanidins are the major polyphenols [85]. Assuming that the decrease of vescalagin is caused by oxidative degradation, vescalagin was treated with polyphenol oxidase in vitro. Although vescalagin content did not decrease after treatment with the enzyme alone, it rapidly decreased when ( +)-catechin, which exists in young leaves, was added to the reaction mixture (Fig. 17A). This is explained by a coupled oxidation mechanism in which the enzyme first oxidizes the catechin B-ring to an *o*-quinone, and the resulting *o*-quinone oxidizes vescalagin (Fig. 1B), accompanied by the production of catechin [10]. The oxidation of vescalagin is regioselective, and the product contains a characteristic cyclopentene-1,2-dione moiety. The presence of this product in young oak leaves was confirmed by HPLC analysis of the quinoxaline derivative produced by treatment with 1,2-phenylenediamine (Fig. 17B). Furthermore, the electrophilic 1,2-dione of the product spontaneously reacts with the catechin A-ring to produce a catechin adduct (Fig. 17C) [85]. These results suggest that the decrease in vescalagin content in oak leaves is caused by oxidation and subsequent coupling with the A-rings of catechin or procyanidins.

Shiitake mushroom (*Lentinula edodes*) is cultivated on the dead logs of *Quercus acutissima*,* Quercus serrata*, *Castanea crenata*, and related trees belonging to Fagaceae. Vescalagin is the major ellagitannin in the wood of these trees. Interestingly, treatment of vescalagin with the mycelia of shiitake mushroom yielded the same oxidation product as that obtained by the above enzymatic oxidation (Fig. 17D) [85]. In this case, the product was further metabolized to yield a complex mixture containing quercusnin A, which has an unusual skeleton [86]. Tannins are produced by plants as a defense mechanism against microorganisms, and Shiitake mushroom is a wood-decay fungi in natural forests; therefore, studies on the degradation mechanisms of tannins in fungi are significant for understanding the ecological role of tannins.

### Fate of ellagitannins in whisky barrels

**Fig. 18:**
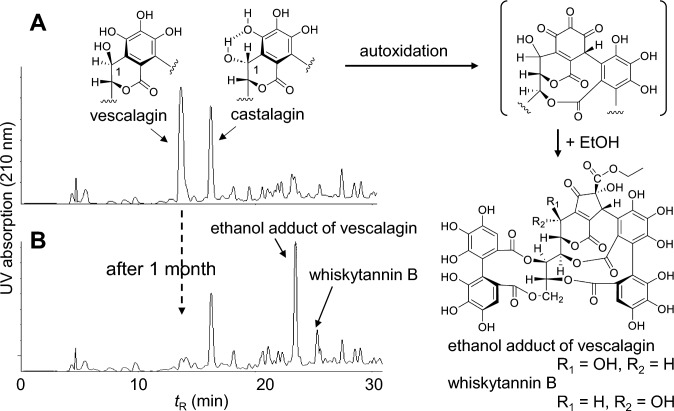
HPLC profile the of 60% EtOH extract of Japanese oak wood (*Quercus crispula*) and production of the ethanol adducts. HPLC after extraction for 1 day (**A**). HPLC after extraction for 1 month (**B**)

Aging distilled spirits in barrels for years is an important process in whisky making. The barrels are made of oak wood, which contains vescalagin and its C-1 epimer, castalagin, as prominent ellagitannins. These ellagitannins are extracted from the barrel into the spirits during aging; however, they are usually not detected in commercial whisky because of their decomposition by autoxidation during the aging process. HPLC analysis of the 60% EtOH extract of Japanese oak wood revealed that vescalagin and castalagin were the major constituents (Fig. 18A). However, after one month, vescalagin was not detected, and an ethanol adduct of a vescalagin autoxidation product was produced (Fig. 18B) [85]. The ethanol adduct of castalagin was identified as whiskytannin B, which was previously isolated from a commercial whisky product as a minor constituent [87]. As observed in the HPLC after extraction for 1 month (Fig. 18B), castalagin was significantly more stable than vescalagin, and its stability was attributed to the formation of intramolecular hydrogen bonds between the hydroxy group at glucose C-1 and a neighboring phenolic group (Fig. 18A) [88]. Tannins in whisky influence mouthfeel, flavor, and color; however, the ethanol adducts of ellagitannin oxidation products are further degraded during the aging process and the degradation products in commercial whisky are not chemically unknown at present.

### Non-extractable ellagitannins in heartwood

**Fig. 19:**
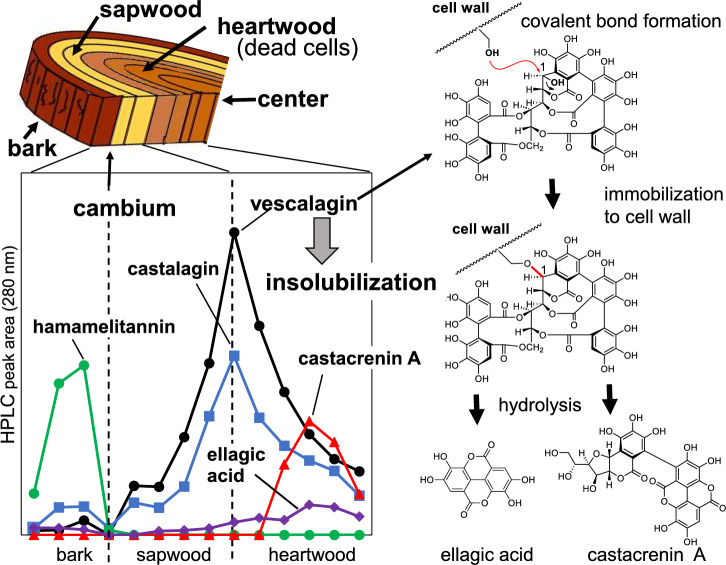
Distribution of tannins in *Castanea crenata* wood and a possible insolubilization mechanism of vescalagin in heartwood. Hamamelitannin: 2′,5-digalloyl hamamelose

Oak barrels are also used for aging wine and brandies; therefore, oak-wood ellagitannins have long been studied. One study reported the presence of non-extractable ellagitannins in the heartwood of European sweet chestnut (*Castanea sativa*) and sessile oak (*Quercus petraea*) [89]. In this context, we studied the distribution of ellagitannins in Japanese chestnut (*Castanea crenata*) wood [90], which is a building material with long-term durability owing to its high tannin content (approximately 8%). Sapwood, the outer layer of wood, contains living cells that can biosynthesize tannins. As the tree grows, the living cells of the inner sapwood die, and heatwood, the inner layers of mature trees, is enlarged; thus, heatwood only comprises dead cells. Vescalagin and castalagin, the major tannins in wood, are mainly biosynthesized in the sapwood-heartwood transitional region, where living cells die off, and tannin concentration decreases in heartwood (Fig. 19). Based on the assumption that the decrease in tannins is due to immobilization in the heartwood, some model experiments were performed [91]. The results showed that the hydroxy group at the benzylic C-1 position of vescalagin undergoes nucleophilic substitution reactions with the hydroxy groups of glycerol and glucose, as well as the double bond of sinapylaldehyde. Therefore, covalent bond formation with cell-wall components is likely responsible for the immobilization of ellagitannins. Furthermore, ellagic acid and castacrenin A are presumed to be generated in inner heartwood by the hydrolysis of immobilized ellagitannins (Fig. 19) [90, 91]. Because ellagitannins display antibacterial activity [92], coating the cell wall with ellagitannins may act as a defense system for heartwood. In addition, whisky barrels are made of oak heartwood, and ellagic acid is a major constituent in whisky.

## Conclusions

This review summarizes the current understanding of the structural changes in tannins associated with food processing and plant growth. The first part addresses the reactions of procyanidins and catechins with electrophiles, which are ascribed to the nucleophilic properties of the A-ring methine carbons. This review also describes a hypothetical mechanism for the production of ellagitannin acyl groups and the degradation and insolubilization of vescalagin. In both these processes, some reactions are non-enzymatic [12, 18, 41], and some enzymatic reactions are chemically replicable without enzymes [77, 82, 93]. The inherent properties of catechins induce similar reactions, even in different situations, as indicated by the similarity in the enzymatic production of thearubigin and the oligomerization of EGCg during autoxidation.

Structural changes in tannins are important for understanding plant ecology and food manufacturing. Although tannins are produced by plants to defend themselves against herbivores, the insolubilization of astringent persimmon proanthocyanidins makes the fruits edible and allows herbivores to disperse the seeds once they have eaten the fruit. The alleviation of the astringency and bitterness of galloyl catechins by enzymatic oxidation has led to the development of black tea. The mechanism for ellagitannin metabolism described in this review is hypothetical and controversial, and further verification using different approaches is necessary. Current knowledge of ellagitannin biosynthesis is very limited, and many questions remain, such as how enzymes regulate regio- and stereospecific coupling between galloyl groups attached to the glucose 3,6- and 2,4-positions of amariin (Fig. 15). The formation of vescalagin from pentagalloyl glucose is another interesting topic that has not been fully investigated (Fig. 17). Thus, many questions regarding the chemistry of tannins remain unresolved.
